# Immunohistochemical detection of p53 and pp53 Ser^392^ in canine hemangiomas and hemangiosarcomas located in the skin

**DOI:** 10.1186/s12917-020-02457-6

**Published:** 2020-07-13

**Authors:** María José García-Iglesias, Jose Luis Cuevas-Higuera, Ana Bastida-Sáenz, María Gracia de Garnica-García, Laura Polledo, Paula Perero, Jorge González-Fernández, Beatriz Fernández-Martínez, Claudia Pérez-Martínez

**Affiliations:** 1 Histology and Pathological Anatomy Section, Department of Animal Health, Faculty of Veterinary Medicine, University of León, León, Spain; 2 Institute of Biomedicine (IBIOMED), University of León, León, Spain; 3 MicrosVeterinaria, León, Spain

**Keywords:** Hemangiomas, Hemangiosarcomas, Skin, Dog, p53, Phospho-p53 Serine^392^, Cell proliferation, Ki-67

## Abstract

**Background:**

p53 protein is essential for the regulation of cell proliferation. Aberrant accumulation of it usually occurs in cutaneous malignancies. Mutant p53 is detected by immunohistochemistry because it is more stable than the wild-type p53. However, post-translational modifications of p53 in response to ultraviolet radiation are important mechanisms of wild-type p53 stabilization, leading to positive staining in the absence of mutation. The aims were: 1) to analyze the immunohistochemical expression of p53 and phospho-p53 Serine^392^ in canine skin endothelial tumours; and 2) to determine if any relationship exists between p53 and phospho-p53 Serine^392^ overexpression and cell proliferation.

**Results:**

p53 and phospho-p53 Serine^392^ immunolabeling was examined in 40 canine cutaneous endothelial tumours (13 hemangiomas and 27 hemangiosarcomas). Their expression was associated with tumour size, hemangiosarcoma stage (dermal versus hypodermal), histological diagnosis and proliferative activity (mitotic count and Ki-67 index). Statistical analysis revealed a significant increase of p53 immunoreactivity in hemangiosarcomas (median, 74.61%; interquartile range [IQR], 66.97–82.98%) versus hemangiomas (median, 0%; IQR, 0–20.91%) (*p* < .001) and in well-differentiated hemangiosarcomas (median, 82.40%; IQR, 66.49–83.17%) versus hemangiomas (*p* = .002). Phospho-p53 Serine^392^ immunoreactivity was significantly higher in hemangiosarcomas (median, 53.80%; IQR, 0–69.50%) than in hemangiomas (median, 0%; IQR, 0.0%) (*p* < .001). Positive correlation of the overexpression of p53 and phospho-p53 Serine^392^ with mitotic count and Ki-67 index was found in the cutaneous vascular tumours (*p* < .001). The Ki-67 index of the hemangiomas (median, 0.50%; IQR, 0–2.80%) was significantly lower than that of the hemangiosarcomas (median, 34.85%; IQR, 23.88–42.33%) (*p* < .001), and that specifically of well-differentiated hemangiosarcomas (median, 24.60%; IQR, 15.45–39.35%) (*p* = .001). Immunolabeling of 18 visceral hemangiosarcomas showed that the p53 (median, 41.59%; IQR, 26.89–64.87%) and phospho-p53 Serine392 (median, 0%; IQR, 0–22.53%) indexes were significantly lower than those of skin (*p* = .001; *p* = .006, respectively).

**Conclusions:**

The p53 and phospho-p53 Serine^392^overexpression together with high proliferative activity in hemangiosarcomas versus hemangiomas indicated that p53 might play a role in the acquisition of malignant phenotypes in cutaneous endothelial neoplasms in dogs. The Ki-67 index may be useful in distinguishing canine well-differentiated hemangiosarcomas from hemangiomas.

## Background

The *p53* tumour oncosuppressor gene (*TP53*) is constitutively expressed in almost all cell types, and it acts as a transcription factor involved in different cellular processes, such as cell cycle control, senescence and apoptosis, differentiation and development, DNA repair, and maintenance of genomic stability [1]. *TP53* gene mutations have been reported in a variety of canine tumours [2–6]. Genetic methods are usually employed to identify these mutations because they are very accurate. However, they are limited by complexity, cost, and collection and storage requirements [7]. Under normal conditions, wild-type (wt) p53 protein (p53) expression is undetectable by immunohistochemistry (IHC) in paraffin-embedded samples due to its short half-life [8]. However, mutations of the *TP53* gene usually cause an abnormal accumulation of aberrant (mutated) p53, which is much more stable and can be detected by IHC [9]. Thus, nuclear immunoreactivity of p53 is generally accepted as an indirect indicator of *TP53* gene mutation [4, 9]. However, post-translational modifications (including multisite phosphorylation and acetylation) of p53 in response to genotoxic and non-genotoxic stresses have been proposed as important mechanisms of wt p53 stabilization and functional regulation, leading to positive staining in the absence of mutation [10, 11]. Thus, cell accumulation of p53 could be the outcome of two circumstances: a) activated p53, which regulates the cell cycle by inducing G1-phase arrest or apoptosis in damaged cells [11]; or b) mutant p53, which may lead to uncontrolled cell growth [12].

DNA damage caused by ultraviolet radiation (UVR) normally activates the *TP53* gene and leads to the accumulation of phosphorylated p53 [13]; Serine^392^ (Ser^392^) residue has been reported as a major UVR-stimulated phosphorylation site [14, 15]. Likewise, UVR can also lead to mutations of the *TP53* gene; its functioning may be prolonged, and cells may proliferate and grow [12]. Chronic exposure to UVR has been proposed as a predisposing factor for the development of canine hemangiomas and hemangiosarcomas (HSAs) in the skin [16, 17]. Results provided to date on p53 in canine endothelial tumours are contradictory [18–22]. Furthermore, most studies analyze HSAs located in viscera, with scarce information available on this type of tumour in the skin.

The aims of this study are: 1) to analyze the immunohistochemical expression of p53 and phospho-p53 Serine^392^ in canine endothelial tumours that are located in the skin; and 2) to determine if any correlation exists between p53 and phospho-p53 Serine^392^ overexpression and cell proliferation in skin tumours.

## Results

### Clinicohistopathological features

Forty dogs with histopathologically confirmed cutaneous endothelial tumours (13 hemangiomas and 27 HSAs) were included in the study. Thirty-two dogs (80%) showed solitary lesions and 8 (20%) had multiple lesions. Six out of 40 dogs (15%) presented other non-vascular neoplasms concomitantly: 4 cases of mammary carcinomas and two mast cell tumours.

The mean age of dogs with hemangioma was 7.22 years (range 3 to 10 years) and there were 7 females, 2 males and 4 missing data. Five breeds were represented, including German shepherd (36.36%, *n* = 4), Boxer (27.27%, *n* = 3), mixed breed dogs (18.18%, *n* = 2), Pug (9.09%, *n* = 1) and Toy Fox Terrier (9.09%, n = 1). This information is missing in 2 cases. Five out of 9 (55.55%) were short-haired dogs. The two mixed breed dogs were not considered because their hair characteristics were not known. There were similar number of tumours in ventral (45.45%, *n* = 5) and non-ventral (54.55%, *n* = 6) body locations. All 13 hemangiomas were located in the hypodermis (Table 1) and had a similar histological appearance, which was characterized by regular and well-defined vascular channels filled with erythrocytes, lined by a single layer of uniform endothelial cells with inconspicuous nuclei. Nuclear pleomorphism ranged from none to mild. Only one out of 12 hemangiomas evaluated showed actinic changes characterized by altered collagen and epidermal dysplasia. Most of the hemangiomas were less than 5 cm in size (Table 1).

**Table 1:** Location within the skin, actinic changes and tumor size of canine cutaneous hemangiomas and hemangiosarcomas

Cutaneous endothelial tumors	Location within the skin ^a^			Actinic changes ^b^			Tumor size ^c^		
	Number of tumors/total tumors (percentage)		***P*** value	Number of tumors/total tumors (percentage)		***P*** value	Number of tumors/total tumors (percentage)		***P*** value
	Dermal	Hypodermal		No	Yes		≤5 cm	> 5 cm	
**Histological diagnosis**^d^			.008			.001			.491
Hemangioma	0/13 (0%)	13/13 (100%)		11/12 (91.67%)	1/12 (8.33%)		10/11 (90.91%)	1/11 (9.09%)	
Hemangiosarcoma	10/25 (40.0%)	15/25 (60.0%)		0/17 (0%)	17/17 (100%)		20/24 (83.33%)	4/24 (16.67%)	
**Histological differentiation scoring**^d^			.667			–			1.000
Well-differentiated	4/8 (50.0%)	4/8 (50.0%)		0/6 (0%)	6/6 (100%)		8/9 (88.89%)	1/9 (11.11%)	
Moderately differentiated	6/17 (35.29%)	11/17 (64.71%)		0/11 (0%)	11/11 (100%)		12/15 (80.0%)	3/15 (20.0%)	

Non-included in inferential statistical study the poorly differentiated hemangiosarcoma (case No. 40)

^a^ Unavailable data in 1 hemangiosarcoma (case No. 22)

^b^ Unavailable data in 1 hemangioma (case No. 2) and 9 hemangiosarcomas (cases Nos. 14, 16, 17, 24, 28, 31, 32, 36, and 37)

^c^ Unavailable data in 2 hemangiomas (cases Nos. 6 and 7) and 2 hemangiosarcomas (cases Nos. 28 and 29)

^d^ Fisher’s exact test

The mean age of dogs with cutaneous HSA was 8.22 years (range 2 to 13 years). The ratio of male to female dogs was 1.36:1 with 57.69% of dogs with HSA being male. Fourteen breeds were recorded in 26 cases, including Boxer (19.23%, *n* = 5), mixed breed dogs (15.38%, *n* = 4), Whippet (7.69%, *n* = 2), Staffordshire bull terrier (7.69%, n = 2), Greyhound (7.69%, n = 2), German shepherd (7.69%, n = 2), Golden Retriever (7.69%, n = 2) and one each of other breeds (26.92%, *n* = 7). Overall, 17 out of 22 dogs (77.27%) were short-haired dogs. The four mixed breed dogs were not considered because their hair characteristics were not known. Where localization was recorded (*n* = 24), 62.5% of tumours occurred in ventral location (ventral chest and abdomen/medial thighs) and 37.5% in non-ventral location (head/lateral chest/limbs). There were 10 dermal HSAs (stage I), 15 hypodermal HSAs (stage II) and 2 missing cases which included cases Nos. 22 and 40 (Table 1). They were primary cutaneous tumours because no visceral HSA was diagnosed concomitantly. Of the 27 HSAs, 9 (33.33%) were well-differentiated, 17 (62.96%) were moderately differentiated and one (3.71%; case No. 40) was poorly differentiated. This latter neoplasm was not included in the inferential statistical analysis because there was only one case. The 17 cutaneous HSAs evaluated for histopathological actinic changes showed mainly altered collagen, dermatitis and epidermal dysplasia (Table 1). Data on actinic changes could not be evaluated in 9 HSAs included in inferential statistical analysis. Most of the HSAs were smaller than 5 cm (Table 1).

The tumour location within the skin and actinic changes were significantly associated with histological diagnosis (hemangioma versus HSA) but not with the histological differentiation scoring (well-differentiated versus moderately differentiated) (Table 1). Likewise, no statistically significant differences were found between the tumour size and the histological diagnosis or the histological differentiation scoring (Table 1).

The mean age of dogs with visceral HSAs was 9.25 years (range 4 to 14 years) and there were 10 males, 5 females and 3 missing data. Eight breeds were represented, including German shepherd (21.43%, *n* = 3), Boxer (21.43%, n = 3), mixed breed dogs (21.43%, n = 3), and one each of other breeds (35.71%, *n* = 5). Breed was missing in 4 cases.

### Evaluation of cell proliferation

**Fig. 1:**
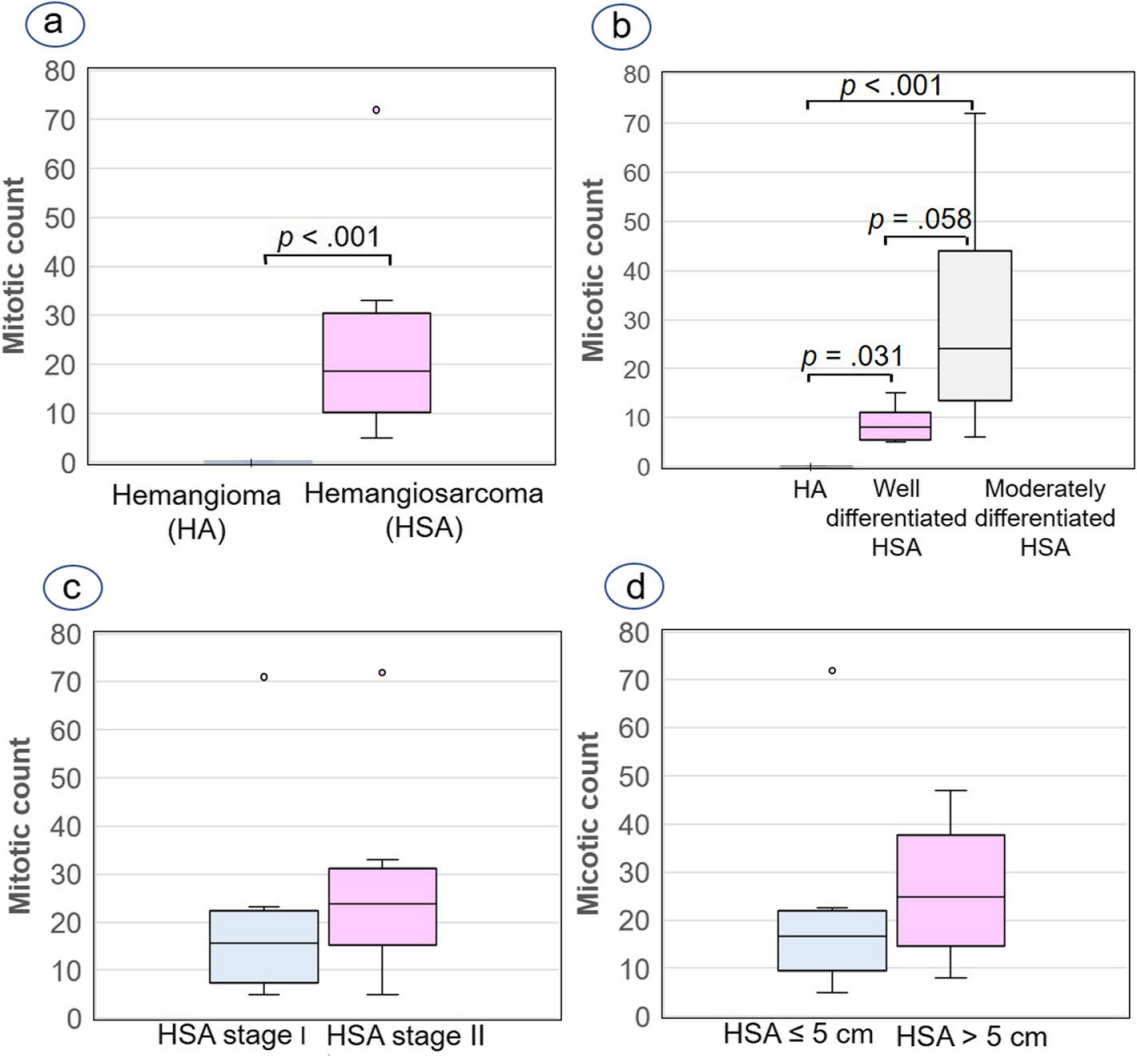
Box plots of median and interquartile range (IQR) mitotic count. **a** Hemangioma (median = 0%; IQR = 0%) versus hemangiosarcoma (HSA) (median = 14.50%; IQR = 8.75–33.0%). **b** Hemangiomas versus well-differentiated HSAs (median = 8.0%; IQR = 5.50–11.0%) versus moderately differentiated HSAs (median = 24.0%; IQR = 13.50–44.0%). **c** HSA stage I (median = 11.50%; IQR = 6.0–23.25%) versus HSA stage II (median = 22.0%; IQR = 13.0–33.0%). **d** HSA size ≤5 cm (median = 13.0%; IQR = 8.25–22.50%) versus HSA > 5 cm (median = 24.0%; IQR = 11.50–41.75%). Mann-Whitney U test (**a**, **c**, **d**) and Mann-Whitney pairwise comparisons (**b**) were applied for statistical analysis

**Fig. 2:**
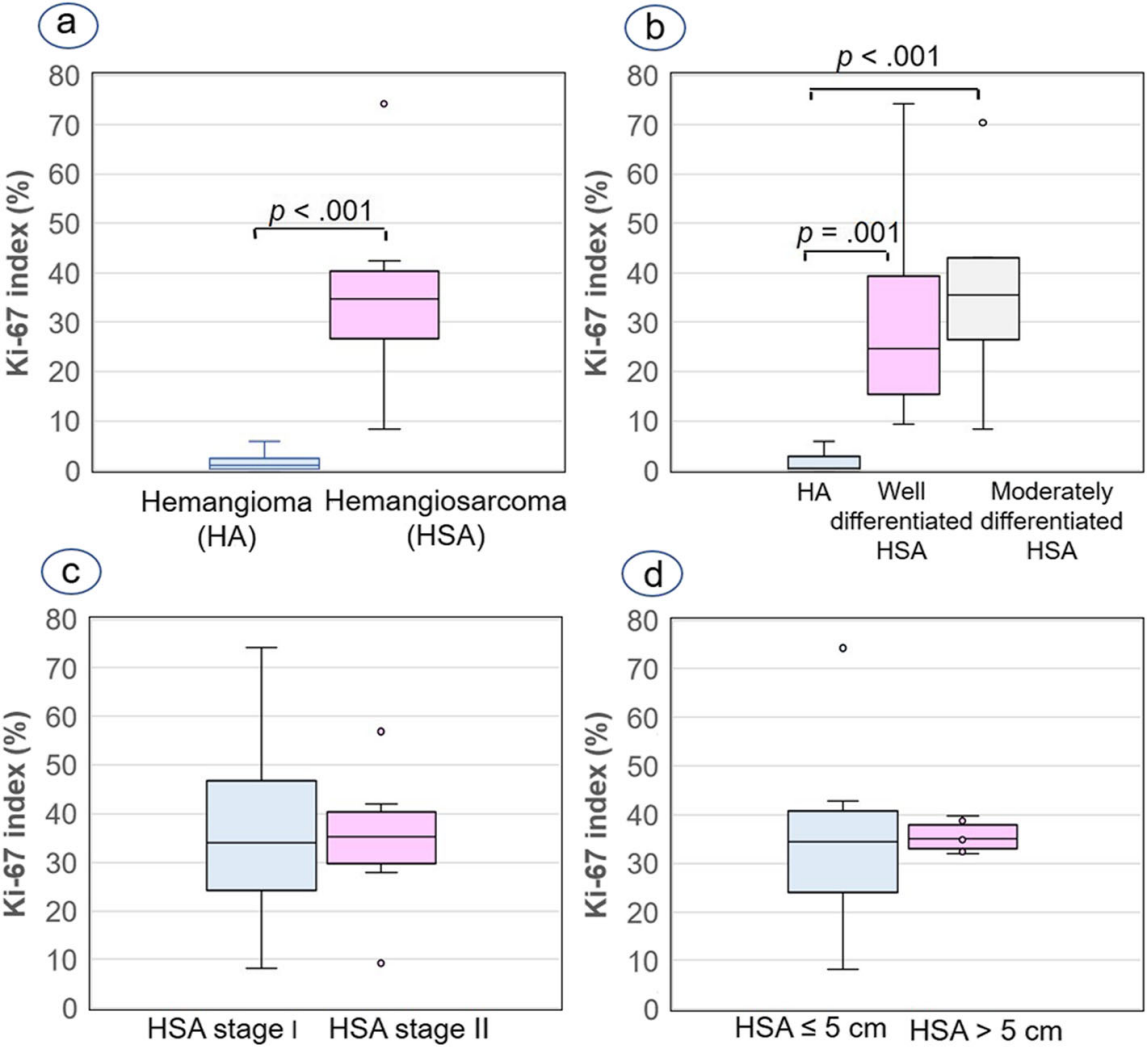
Box plots of median and interquartile range (IQR) Ki67 index. **a** Hemangioma (median = 0.50%; IQR = 0–2.80%) versus hemangiosarcoma (HSA) (median = 34.85%; IQR = 23.88–42.33%). **b** Hemangioma versus well-differentiated HSAs (median = 24.60%; IQR = 15.45–39.35%) versus moderately differentiated HSAs (median = 35.60%; IQR = 26.50–43.10%). **c** Stage I HSAs (median = 31.40%; IQR = 21.73–50.03%) versus stage II HSAs (median = 35.60%; IQR = 27.90–42.10%). **d** HSA size ≤5 cm (median = 34.20%; IQR = 20.58–42.93%) versus HSA > 5 cm (median = 34.85%; IQR = 32.45–38.75%). Mann-Whitney U test **a**, Mann-Whitney pairwise comparisons (**b**) and Student t test (**c**, **d**) were applied for statistical analysis

**Fig. 3:**
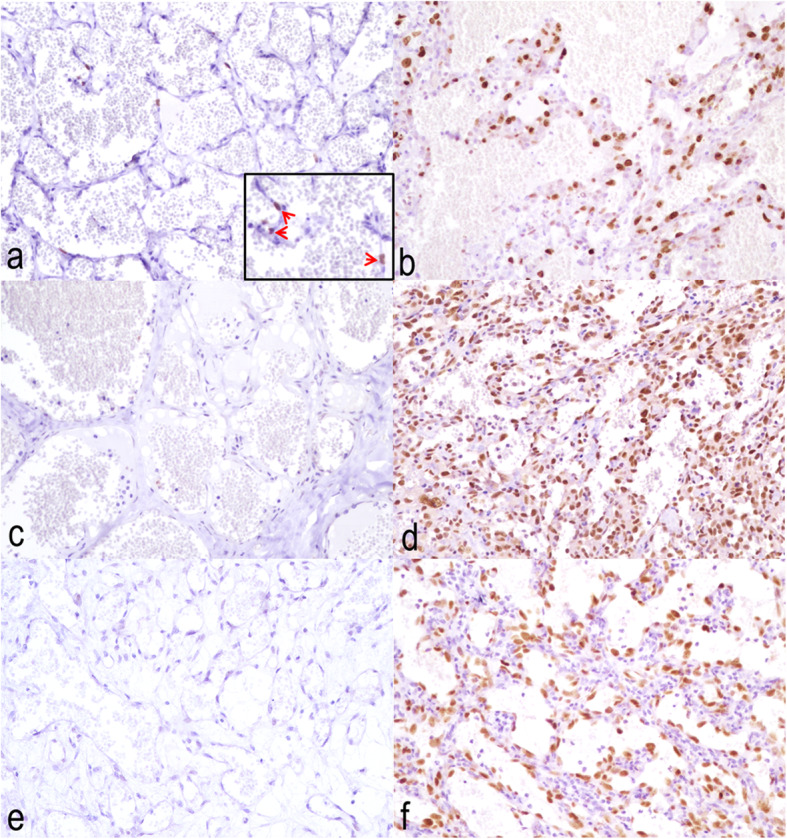
Immunohistochemical expression of proliferative and cell cycle regulatory markers. **a** Hemangioma. Presence of few Ki-67-positive tumour nuclei. Inset: Positive nuclear expression (arrows). **b** Well-differentiated hemangiosarcoma. Numerous Ki-67-positive neoplastic nuclei. **c** Hemangioma. No labeling for p53. **d** Well-differentiated hemangiosarcoma. Note a high number of neoplastic cells showing strong nuclear expression for p53. **e** Hemangioma. No labeling for pp53 Ser^392^. **f** Moderately differentiated hemangiosarcoma. Strong nuclear pp53 Ser^392^ reaction in numerous neoplastic cells. Peroxidase-DAB revelation system, Harris Haematoxylin counterstain. 200x

No mitotic figure was observed in hemangiomas (mitotic count, MC = 0), while the MC in HSAs varied between 5 and 72 mitoses in 10 high-power fields (400x), with a range of 5–15 mitotic figures in well-differentiated tumours and 6–72 mitoses in moderately differentiated neoplasms. The MC in the poorly differentiated HSA (case No. 40) was 38 (see Additional file 1). Statistical analysis revealed that the MC in HSAs was significantly higher than in hemangiomas (Fig. 1a) Significant differences were also shown between well- and moderately differentiated HSAs versus hemangiomas and there was a clear trend between well and moderately differentiated HSAs (Fig. 1b). The MC was not significantly associated with the HSA stage (Fig. 1c) or HSA size (Fig. 1d). The Ki-67 index ranged from 0 to 5.90% in hemangiomas and from 8.30 to 74.30% in HSAs located in skin (see Additional file 1). With reference to histological differentiation scoring, Ki-67 index ranged from 9.20 to 74.30% in well-differentiated HSAs and from 8.30 to 70.50% in moderately differentiated HSAs. This proliferation index was 28.10% in the poorly differentiated HSA (case No. 40, see Additional file 1). The percentages of Ki-67-positive cells were significantly lower in hemangiomas than in HSAs (Fig. 2a). Similar to the MC, the Ki-67 index was lower in hemangiomas than in well- and moderately differentiated HSAs, while no significant differences were found between well-differentiated and moderately differentiated HSAs (Figs. 2b, 3a and b). The Ki-67 index was not significantly associated with the HSA stage (Fig. 2c) or HSA size (Fig. 2d).

### Evaluation of cell cycle regulatory p53 expression

Most of the hemangiomas (11/13; 84.62%) showed < 38% of p53-positive immunoreactivity, while > 38% of p53-positive nuclei was only observed in 2/13 (15.38%) hemangiomas (see Additional file 1), in which cell proliferation was always low (MC = 0 and Ki-67 < 6%).

**Fig. 4:**
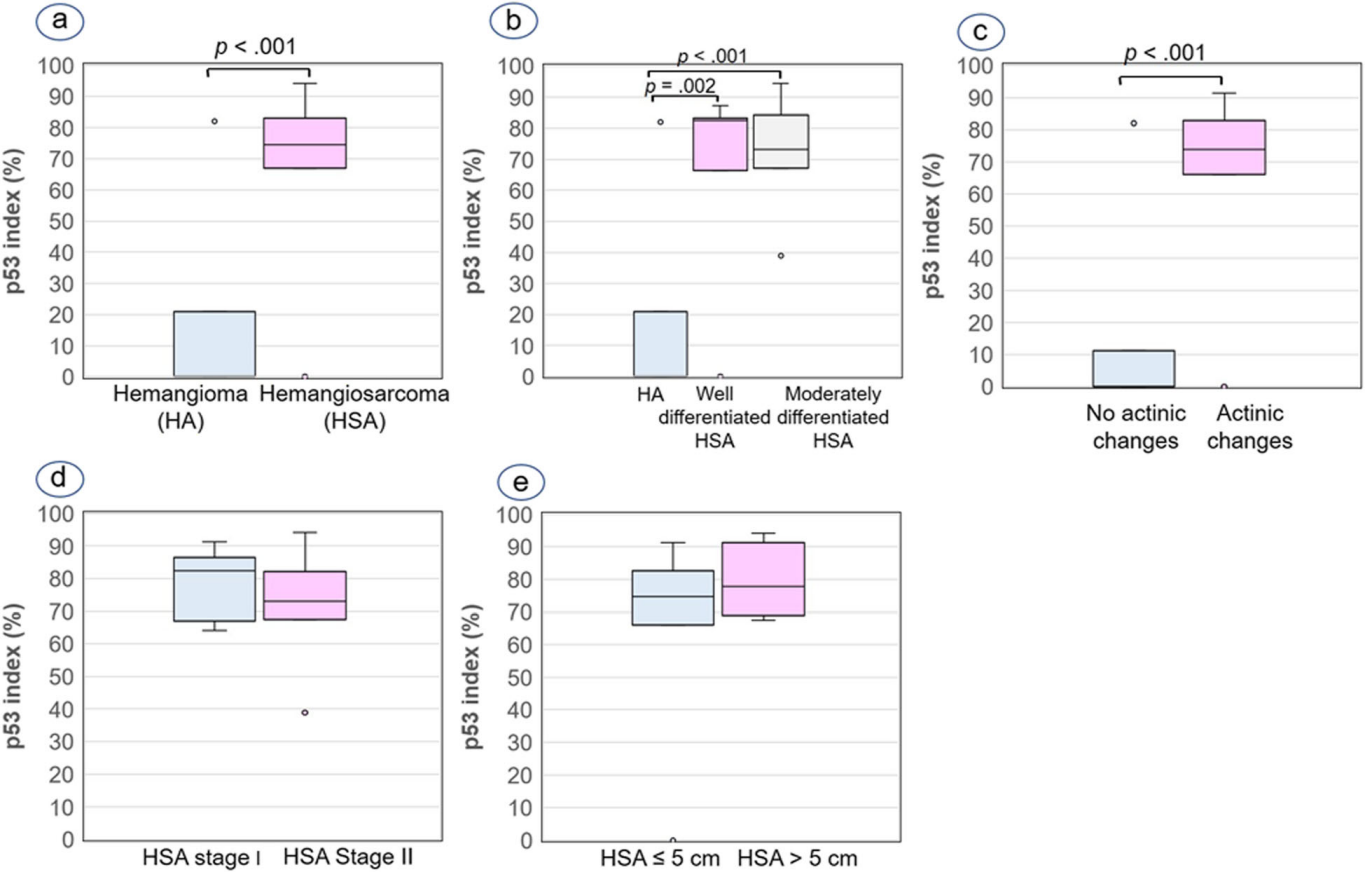
Box plots of median and interquartile range (IQR) p53 index. **a** Hemangioma (median = 0%; IQR = 0–20.91%) versus hemangiosarcoma (HSA) (median = 74.61%; IQR = 66.97–82.98%). **b** Hemangiomas versus well-differentiated HSAs (median = 82.40%; IQR = 66.49–83.17%) versus moderately differentiated HSAs (median = 73.06%; IQR = 67.11–84.32%). **c** Cutaneous vascular tumours without actinic changes (median = 0%; IQR = 0–11.17%) versus with actinic changes in the skin (median = 73.94%; IQR = 66.05–82.83%). **d** Stage I HSAs (median = 82.33%; IQR = 66.97–86.77%) versus stage II HSAs (median = 73.06%; IQR = 67.53–82.40%). **e** HSA size ≤5 cm (median = 74.61%; IQR = 66.11–82.54%) versus HSA > 5 cm (median = 77.93%; IQR = 68.91–91.39%). Mann-Whitney U test (a, c,e), Mann-Whitney pairwise comparisons(**b**) and Student t test (**d**) were applied for statistical analysis

Most of the skin HSAs (25/27, 92.6%) showed a p53 index ranging from 38.84 to 94.25%, except for 2 cases (case Nos. 22 and 40), in which no immunolabeling for p53 was seen (see Additional file 1). Statistical analysis demonstrated a significant relationship between the p53 index and the tumour diagnosis owing to a lower immunolabeling of p53 in hemangiomas than in skin HSAs (Fig. 4a). Similar to both markers for cell proliferation, the p53 index was significantly lower in hemangiomas than in well- and moderately differentiated HSAs but no differences were observed between well- and moderately differentiated HSAs (Figs. 3c and d; 4b). In addition, the p53 index was significantly higher in the 18 vascular tumours (1 hemangioma and 17 HSAs) with actinic changes (median, 73.94%; IQR, 66.05–82.83%) than in 11 hemangiomas without these histological changes (median, 0.0%; IQR, 0.0–11.17%; Fig. 4c). However, no relationship between p53 index and HSA stage (Fig. 4d) or HSA size (Fig. 4e) was found.

**Fig. 5:**
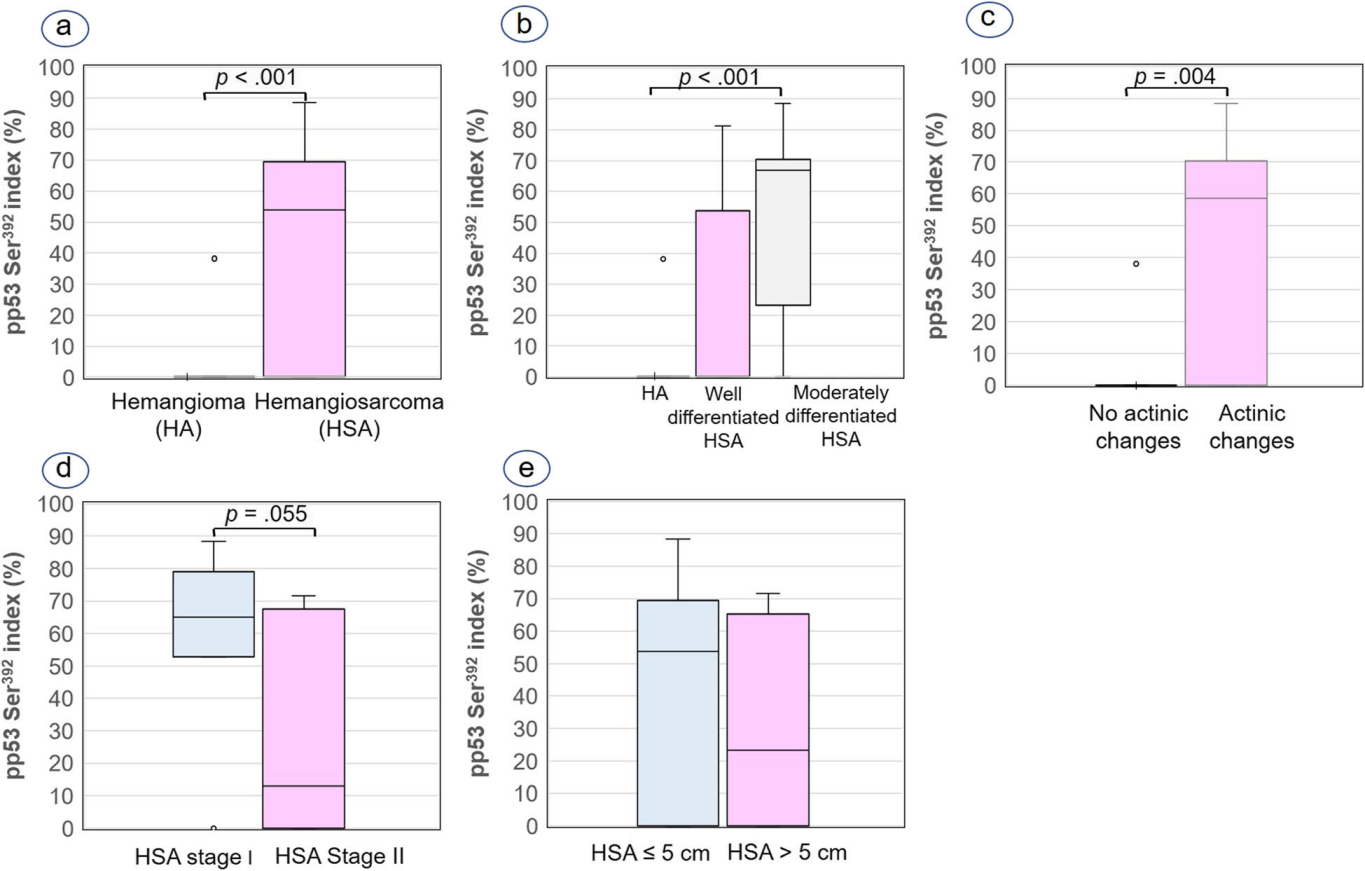
Box plots of median and interquartile range (IQR) pp53 Ser392 index. **a** Hemangioma (median = 0%; IQR =0%) versus hemangiosarcoma (HSA) (median = 53.80%; IQR = 0–69.50%). **b** Hemangiomas versus well-differentiated HSAs (median = 0%; IQR = 0–53.65%) versus moderately differentiated HSAs (median = 66.90%; IQR = 23.35–70.50%). **c** Cutaneous vascular tumours without actinic changes (median = 0%; IQR = 0%) versus with actinic changes in the skin (median = 58.70%; IQR = 0–70.30%). **d** Stage I HSAs (median = 65.15%; IQR = 52.78–79.65%) versus stage II HSAs (median = 12.80%; IQR = 0–67.90%). **e** HSA size≤5 cm (median = 53.80%; IQR = 0–69.33%) versus HSA > 5 cm (median = 23.35%; IQR = 0–65.30%). Mann-Whitney U test (**a**, **c**, **d**, **e**) and Mann-Whitney pairwise comparisons (**b**) were applied for statistical analysis

No pp53 Ser^392^-positive tumour cells were observed in 12/13 hemangiomas (92.31%). Only the case No. 8 (1/13; 7.69%) presented 38.20% positive tumour cells, though with low cell proliferation (MC = 0 and Ki-67 index = 2.70%) (see Additional file 1). Seventeen out of 27 HSAs (62.96%) showed a pp53 Ser^392^ index ranging 12.81 to 88.5% while no immunolabeling for pp53 Ser^392^ was observed in the other skin HSAs (see Additional file 1). Immunoreactivity for pp53 Ser^392^ was significantly higher in HSAs than in hemangiomas (Figs. 3e and f; 5a). However, unlike both proliferative markers and the p53 index, differences were only demonstrated between hemangiomas and moderately differentiated HSAs for pp53 Ser^392^ expression (Fig. 5b). Similar to the p53 index, the pp53 Ser^392^ index was significantly higher in the 18 cutaneous tumours (1 hemangioma and 17 HSAs) with actinic changes (median, 58.70%; IQR, 0.0–70.30%) than in the 11 hemangiomas without these histological changes (median, 0.0%; IQR, 0.0%; Fig. 5c). On the other hand, there was also a borderline significant association (*p* = .055) between the pp53 Ser^392^ labeling index and HSA stage, being higher in stage I than stage II (Fig. 5d). No statistical differences were found between the pp53 Ser^392^ index and HSA size (Fig. 5e).

**Fig. 6:**
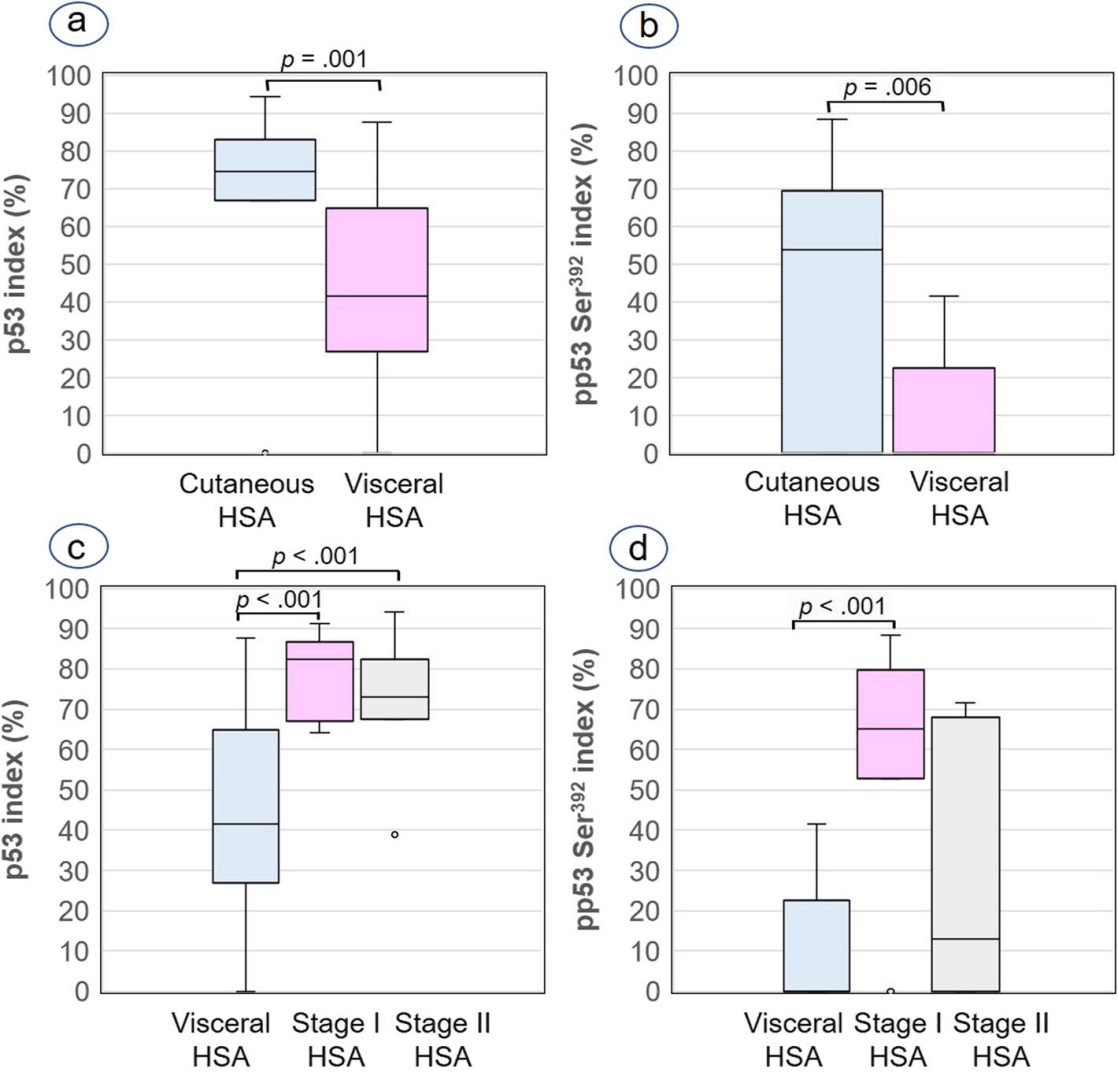
Box plots of median and interquartile range (IQR) p53 and pp53 Ser392 indexes in relation to body location of HSAs. **a** p53 index. Cutaneous HSAs (median = 74.61%; IQR = 66.97–83.98%) versus visceral HSAs (median = 41.59%; IQR = 26.89–64.87%). **b** pp53 Ser^392^ index. Cutaneous HSAs (median = 53.80%; IQR = 0–69.50%) versus visceral HSAs (median = 0%; IQR = 0–22.53%). **c** p53 index. Visceral HSAs versus stage I/dermal HSAs (median = 82.33%; IQR = 66.97–86.77%) versus stage II/hypodermal (median = 73.06%; IQR = 67.53–82.40%). **d** pp53 Ser^392^ index. Visceral HSAs versus stage I/dermal HSAs (median = 65.15%; IQR = 52.78–79.65%) versus stage II/hypodermal HSAs (median = 12.80%; IQR = 0–67.90%). Mann-Whitney U test (**a**, **b**), Tukey test (**c**) and Mann-Whitney pairwise comparisons (**d**) were applied for statistical analysis

The visceral HSAs (16/18; 88.8%) showed a p53 index ranging from 17.71 to 87.6%, and no immunolabeling for p53 was observed only in 2 cases (case Nos. 8 and 9) (see Additional file 2). Besides, 8 out of 18 visceral HSAs (44.44%) showed pp53 Ser^392^ immunolabeling ranging from 12.3 to 41.5% (see Additional file 2). The p53 and pp53 Ser^392^ indexes were significantly lower in visceral HSAs than cutaneous HSAs (Fig. 6a and b, respectively). Other interesting result was that p53 index was significantly higher in stage I and II HSAs than in visceral HSAs (Fig. 6c), and a significant higher pp53 Ser^392^ index was also demonstrated in HSAs situated in dermis (stage I) than in visceral tumours (Fig. 6d).

### Correlation between proliferation and cell cycle regulatory proteins

Cell proliferation markers (MC and Ki-67 index) and cell cycle regulatory proteins (p53 and pp53 Ser^392^) were significantly related to each other in the cutaneous vascular tumours examined (hemangiomas and HSAs). The MC was positively related to the Ki-67 index (ρ = .753; *p* < .001); and the p53 index was correlated to the pp53 Ser^392^ index (ρ = .688; *p* < .001). There was also a positive correlation between the MC and the immunoreactivity for p53 (ρ = .600; *p* < .001) and pp53 Ser^392^ (ρ = .558; *p* < .001). In addition, the Ki-67 index was positively related to the p53 index (ρ = .667; *p* < .001) and the pp53 Ser^392^ index (ρ = .533; *p* < .001).

## Discussion

In the present study, immunohistochemical expression of p53 and pp53 Ser^392^ was analyzed in canine cutaneous endothelial tumours. The patient characteristics with hemangiomas were similar to some previous publications, with mean age of diagnosis below 7.5 years and female predisposed [23], but different to others with higher mean age and no sex predilection [24]. In dogs with skin HSAs, the epidemiological data concerning the mean age of animals (8.22 years) and the lack of sex predilection were consistent with previous reports on cutaneous HSAs [25, 26]. The high frequency of shorthaired breeds (Boxer, Whippet, Greyhound, and Staffordshire bull terrier) and the ventral location of tumours in this study were similar to the findings from other studies which suggest solar-induced lesions [26, 27]. Nevertheless, these findings should be interpreted cautiously due to the relatively small size of the population examined.

Current data on the role of the *TP53* oncosuppressor gene during the development of canine cutaneous vascular neoplasms are scarce and controversial [18, 19, 22]. Consistent with reports on human angiosarcoma [28, 29], the results of the present canine study showed a significantly lower IHC expression of p53 in cutaneous hemangiomas than in HSAs; such differences were also found between hemangiomas and well-differentiated HSAs. In addition, the p53 immunolabeling was significantly higher in cutaneous HSAs than in visceral HSAs. These findings suggest that p53 might play a more important role in the development of malignant phenotypes in this type of neoplasm in the skin than in visceral location, and the results also support those seen in earlier studies that indicate that mutation in this gene might contribute to the development of some cases of canine HSAs [18, 22]. Previous studies have found expression of p53 from 0 to 50% of canine HSAs [18, 19, 21, 30]. These data are lower than the 92.6% found for positive cutaneous tumours in the present study. This variability may be attributed in part to the application of different protocols and/or to interpretation of results. Some studies have explored the importance of establishing immunohistochemical cutoff values for the p53 positivity as an indirect indicator to distinguish wt from mutant p53, obtaining different results. Many immunohistochemical studies define a mutant protein as occurring when neoplasms show a nuclear immunoreaction for p53 in more than 10% of the tumour cells [4, 18, 21], while other studies chose a rate of more than 20% [29, 31] or 40% [28, 32, 33] of the tumour cells as their cutoff value. The present study supports a p53 index > 38% as cutoff value because it was found in the 92.6% of tumours which were diagnosed as cutaneous HSAs based on the abnormal proliferation of cells and their histological characteristics of malignancy. This value is very similar to that established to consider the *TP53* gene status as mutated in human angiosarcomas [28]. However, caution must be exercised when interpreting p53 immunostaining because post-translational modifications of p53 in response to various genotoxic and non-genotoxic stresses have been proposed as important mechanisms of p53 stabilization and functional regulation, leading to positive staining in the absence of mutation [11]. Thus, UVR exposure, considered a risk factor in the development of canine cutaneous HSAs, rapidly activates p53 by phosphorylation on several residues and leads to the accumulation of p53 [13], being Ser^392^ reported as a major UVR-stimulated phosphorylation site [14, 15]. In this sense, the high p53 expression observed in the cutaneous HSAs could be also related to p53 stabilization by UVR-stimulated phosphorylation site of Ser^392^ as suggested by a significant higher pp53 Ser^392^ expression in the cutaneous HSAs and, within of them, in the dermal HSAs respect to the visceral HSAs. This hypothesis was also supported by a significant increase of immunolabeling of both markers in the 17 cutaneous HSAs and 1 hemangioma which presented actinic changes respect to 11 hemangiomas without actinic changes. For this reason, it was worth asking if the high p53 expression seen in cutaneous HSAs might be caused by stabilization of the wt p53 or the presence of mutant protein, because the CM1 antibody applied recognizes both wt and various mutant forms of p53 [4].

A useful tool concerning immunohistochemical detection of wt or mutant protein may be the assessment of proliferative activity together with the p53 index, taking in account that accumulation of activated p53, under normal circumstances, regulates the cell cycle by inducing G1-phase arrest or apoptosis in cells that are genetically damaged by UVR or chemical carcinogens, while mutant p53 may lead to uncontrolled cell growth [12]. Besides, evaluation of the coexpression of Ki-67 as a proliferative marker and p53 protein is also supported by the correlation that has been found between both markers in several types of cancer in humans [34] and in some tumours in animals [35]. Based on these previous findings, the significant positive correlation between p53 and the Ki-67 index found in the present study seems to support that alterations in p53 may lead directly or indirectly to increased cellular proliferation in these malignant lesions and the high p53 expression in HSAs is mainly due to the presence of aberrant p53. Thus, the coexpression of p53 with high Ki-67 immunolabeling may be an indicator of tumour progression in canine cutaneous endothelial tumours, as has been described in other tumours [36–40].

The higher pp53 Ser^392^ index in the 17 cutaneous HSAs with actinic changes as well as in dermal HSAs respect to hypodermal and visceral HSAs suggests the Ser^392^ phosphorylation in mutant p53 by UVR exposure. Previous studies in vitro [41] and in human tumours in vivo [42, 43] have also described mutant p53 with multiple p53 phosphorylation sites, including Ser^392^. Besides, a correlation between Ser^392^ phosphorylation in mutant p53 protein and high levels of Ki-67 staining has been found in human carcinoma [43]. This relationship seems to support that Ser^392^ phosphorylation in mutant p53 may contribute to tumor progression [43, 44]. In the same way, the significant positive correlation between pp53 Ser^392^ and Ki-67 expression in cutaneous HSAs but not in hemangiomas might indicate that this post-translational modification may also play a role in the oncogenic function of p53 in canine cutaneous endothelial tumours, although the design of this study cannot confirm this hypothesis. It has been also indicated that the role of Ser^392^ phosphorylation in mutant p53 stability and its oncogenic activity may be dependent on types of *TP53* gene mutations and cellular contexts [45]. Thus, Ser^392^ phosphorylation in mutant p53 could enhance tetramer formation of mutant p53, which may enhance hetero-oligomerization with wt p53 showing the dominant-negative effects and oncogenic gain-of-functional in carcinomas of the urinary tract [46]. However, in vitro studies have shown that Ser^392^A mutation in two hotspot *TP53* mutants (R175H and R248W) transforms rat embryonic fibroblasts in cooperation with Ha-Ras oncogene more potently than R175H and R248W *TP5*3 mutants, indicating that the non-phosphorylated form of mutant p53 at Ser^392^ seems to increase oncogenic activity. In addition, Ser^392^ non-phosphorylatable p53 mutants also had an enhanced ability to confer cellular resistance to the cytotoxic effect of cisplatin [41]. Further studies are needed to confirm the role of p53 and pp53 Ser^392^ in canine cutaneous HSAs and its potential role in the response to co-treatments for surgery or alternative treatments proposed to avoid mutilating surgeries such as photodynamic therapy [47], taking into account that p53 is required for PDT-mediated apoptosis [48]. Finally, based on the results of the current study, a diagnostic use of the Ki-67 index is not only that it may be able to distinguish canine hemangiomas from HSAs owing to significantly higher Ki-67 expression in HSAs than in their benign counterparts, as other studies have described [24], but also that it may distinguish hemangioma from well-differentiated HSAs, as has been described in the case of human angiosarcomas [49]. This use of Ki-67 as an ancillary diagnostic tool is also described in previous studies on canine mammary tumours [3] and melanocytic tumours [50].

## Conclusions

The high expression of p53 and pp53 Ser^392^ in canine cutaneous HSAs, together with high proliferative activity, suggests that the *TP53* gene may play a role in the development of some cases of these cutaneous tumours. These molecules should be further investigated as potential therapeutic targets for cutaneous HSAs. The Ki-67 index may be useful in distinguishing canine well-differentiated hemangiosarcomas from hemangiomas.

### Study limitations

First, all hemangiomas analyzed were located in the hypodermis. We consider the inclusion of dermal hemangiomas to be necessary. Second, only one poorly differentiated HSA was studied, and it was not included in the inferential statistical analysis. It is necessary to increase the number of HSAs and to obtain more poorly differentiated HSAs to analyze the expression of p53 and pp53 Ser^392^ in comparison to well-differentiated and moderately differentiated HSAs. Third, this study is retrospective, and only paraffin embedding tumour tissues were available making it difficult to use genetic methods.

## Methods

### Case selection

A retrospective study was performed on previously diagnosed cutaneous canine endothelial tumours provided by the diagnostic pathology service of the School of Veterinary Science of León and the MicrosVeterinaria laboratory (Spain). Clinical data, pathology reports and paraffin-embedded blocks were collected. Anatomic location of tumour development was divided into ventral (including ventral chest and abdomen/medial thighs) and other locations (head, lateral chest and limbs). This categorization was based on previous studies which suggest that glabrous skin of the ventral body region is especially prone to solar-induced damage [16, 51]. Haematoxylin and eosin-stained (HE) sections were reexamined independently by two veterinary pathologists to confirm the diagnosis, and additional immunohistochemical staining for CD31 was used to confirm endothelial origin. Thirteen hemangiomas and 27 HSAs were diagnosed. Classification of HSAs based on their location within the skin was established following the staging protocol previously described [25]: stage I, a primary tumour confined to the dermis and defined as a dermal tumour; stage II, a primary tumour involving the hypodermis, with or without concurrent dermal involvement and without underlying muscular involvement, defined as a hypodermal tumour; and stage III, any primary tumour with underlying muscular involvement which was defined as a deep tumour. Besides, HSAs were graded for overall differentiation and nuclear pleomorphism following criteria previously established for both cutaneous and visceral HSAs [24]: well-differentiated HSA characterized by numerous, irregular, anastomosing vascular channels and minimal variation in nuclear size and shape; moderately differentiated HSA, with at least 50% of the tumour showing well-defined vascular spaces and a moderate variation in nuclear size and shape; and poorly differentiated HSA presenting few distinct tumour vascular channels and a marked variation in nuclear size and shape.

The number of mitoses per 10 high-power fields (40x objective, 10x ocular, field diameter 0.55 mm) was counted, and the MC was classified into 4 categories [52]: 0 ≤ 10; 1 = 11 to 20; 2 = 21 to 30; and 3 ≥ 30.

Actinic changes were assessed by evaluating for the presence of solar elastosis, ischaemic altered collagen, dermatitis, superficial dermal fibrosis and epidermal dysplasia [27]. These changes were assessed in 17 out of 26 HSAs and 12 out of 13 hemangiomas included in the statistical study.

Eighteen visceral HSAs were included in this study to carry out a comparative immunohistochemical evaluation with cutaneous HSAs and to assess the possible effects of UVR on the phosphorylation of p53 in cutaneous HSAs.

### Immunohistochemistry

Serial 3 μm sections of paraffin-embedded samples of each neoplasm were cut and mounted on poly-L-lysine-coated slides (Thermo Scientific, Germany). Sections were dewaxed in xylene and rehydrated in graded alcohol solutions, and they were stained using the Avidin-Biotin complex method or the EnVision(+) method (Table 2). Endogenous peroxidase activity was quenched by incubation in hydrogen peroxide (0.5%) solution in water for 30 min at room temperature. Pressure-cooker-based antigen retrieval was performed for 3 min at full pressure in 10 mmol/L sodium citrate buffer (pH 6.0) and allowed to cool in the buffer for 20 min at room temperature. Sections were incubated for 14 to 18 h at 4 °C in a humidified chamber with the primary antibodies shown in Table 2. Specific and validated formalin-fixed, paraffin-embedded positive controls were used for each antibody: p53 and pp53 Ser^392^ immunoreactive canine mammary tumour for p53 and pp53 Ser^392^ and canine intestine for Ki-67. The primary antibody was replaced with antibody diluent for negative controls. Labeling was visualized with application of 3–3′-diaminobenzidine-tetrahydrochloride (DAB) as chromogen substrate (Vector Laboratories, Burlingame, CA, USA). Slides were counterstained with Harris hematoxylin, dehydrated in graded alcohol, and mounted with coverslips. Specificity of the p53 clone CM1 polyclonal antibody in dogs has previously been verified [53].

**Table 2:** Antibodies used in immunohistochemical analysis

Antigen	Clone	Source	Dilution	Type of Antibody	Detection System/Source
CD31	JC/70A	DAKO, Copenhagen, Denmark	1:100	Mouse MAb	Vectastain Elite, ABC Kit; Vector Laboratories, Burlingame, California, USA
Ki67	MIB-1	DAKO, Copenhagen, Denmark	1:100	Mouse MAb	Vectastain Elite, ABC Kit; Vector Laboratories, Burlingame, California, USA
p53	CM1	Signet Laboratories, Dedham, MA	1:200	Rabbit PAb	EnVision+ System Labelled Polymer-HRP anti-rabbit (Dako, Copenhagen, Denmark)
pp53 Ser^392^	sc-56,173	Santa Cruz Biotechnology Inc., Santa Cruz, CA	1:100	Mouse MAb	Vectastain Elite, ABC Kit; Vector Laboratories, Burlingame, California, USA

*MAb* monoclonal antibody, *PAb* polyclonal antibody

### Assessment of immunostaining

The presence of brown precipitate in the nucleus, regardless of its intensity, was taken as positivity for p53, pp53 Ser^392^ and Ki-67 [22, 28]. Cell counting was independently performed without prior knowledge of the histopathological diagnosis. The interobserver variability was low. Any discordant interpretation was resolved among the reviewing researchers at a multiheaded microscope. Areas with higher positivity were selected for this quantitative analysis of the immunostaining. The immunoreaction was evaluated using light microscopy (Eclipse E600, Nikon, Japan) with a Nikon Digital Sight DS-Fi1® camera (Nikon, Japan). Approximately 1000 cells were counted at 400x magnification by the manual count tool of the NIS-Elements BR software (Nikon Instruments Inc., Japan). The labeling index was estimated as the number of positive nuclei divided by the total number of nuclei scored, and it was expressed as a percentage.

The immunohistochemical cutoff value for p53 positivity was established using the lowest percentage of positive nuclei obtained in HSAs (38%). This cutoff value is similar to that used in human angiosarcomas [28]. Tumours with values above this cutoff value were considered to be p53-positive.

### Statistical analysis

Descriptive and inferential statistics were used to provide information on the role which cell cycle regulatory p53 and pp53 Ser392 proteins plays in canine cutaneous hemangiomas and HSAs, as well as on its relationship with proliferative activity (MC and Ki-67 index) and clinicohistopathological parameters. Canine visceral HSAs were also analyzed to evaluate differences in p53 and pp53 Ser392 expression with cutaneous HSAs. Data sets were tested for normality using the Shapiro-Wilk test. Each quantitative variable was compared according to the diagnosis (hemangioma versus HSA), histological differentiation scoring (well-differentiated versus moderately differentiated), location of HSA within the skin (dermal/stage I versus hypodermal/stage II tumour) and HSA size (≤5 cm and > 5 cm). To compare groups, the Student t test or Mann-Whitney U test was used when data distribution was normal or non-normal, respectively. Non-parametric Kruskal-Wallis test and, when it was inferior to 0.05, Mann-Whitney pairwise comparisons were applied to analyze differences in mitotic count, Ki-67 index, p53 index and pp53 Ser392 index between hemangioma, well-differentiated HSA and moderately differentiated HAS. ANOVA with post-hoc Tukey test or non-parametric Kruskal-Wallis test and Mann-Whitney pairwise comparisons were used to evaluate differences in p53 and pp53 Ser392 indices between visceral HSAs and cutaneous HSAs (stage I and stage II). The results were expressed as medians and interquartile ranges.

Comparison of tumour size and location within the skin with diagnosis and histological differentiation scoring was performed using Fisher’s exact test. Results for qualitative data were expressed in percentages. Spearman rank correlation (Spearman’s correlation coefficient, ρ) was used to estimate the correlations between mitotic count, Ki-67, p53, and pp53 Ser^392^. Data were analyzed with SPSS version 24 (SPSS, Inc., IBM, Chicago, IL, USA). Significant differences were considered at *p* values < .05, while differences at *p* < .10 were described as tendencies.

## Supplementary information

**Additional file 1 : Table S1.** Histologic grade, mitotic count and Ki-67, p53 and pp53 Ser392 indexes in canine cutaneous hemangiomas and hemangiosarcomas.

**Additional file 2 : Table S2.** p53 and pp53 Ser392 indexes in canine visceral hemangiosarcomas.

## Data Availability

The datasets used and/or analysed during the current study are available from the corresponding author on reasonable request.

## Abbreviations

IQR: Interquartile range
*TP53*: p53 tumour oncosuppressor gene
p53: p53 protein
wt: wild-type
IHC: Immunohistochemistry
UVR: Ultraviolet radiation
Ser^392^: Serine^392^
HSA: Hemangiosarcoma
MC: Mitotic count
HE: Haematoxylin and eosin
DAB: 3–3′-diaminobenzidine-tetrahydrochloride
