# Supplementary material for: Immunohistochemical detection of p53 and pp53 Ser392 in canine hemangiomas and hemangiosarcomas located in the skin
Source: BMC Vet Res. 2020 Jul 13;16:239. doi: 10.1186/s12917-020-02457-6 (PMC7359283; doi:10.1186/s12917-020-02457-6)
Supplement: Supplementary file 1 — Additional file 1 : Table S1. Histologic grade, mitotic count and Ki-67, p53 and pp53 Ser392 indexes in canine cutaneous hemangiomas and hemangiosarcomas. [file 12917_2020_2457_MOESM1_ESM.docx]

**Supplemental Table S1.** Histologic grade, mitotic count and Ki-67, p53 and pp53 Ser^392^ indexes in canine cutaneous hemangiomas and hemangiosarcomas

| **Cases** | **Histologic grade** | **Mitotic count** | **Ki-67 index** | **p53 index** | **pp53 Ser^392^ index** |
| --- | --- | --- | --- | --- | --- |
|  |  | **No. mitosis/10 HPF** | **(%)** | **(%)** | **(%)** |
| No. 1 | - | 0 | 0.50 | 0 | 0 |
| No. 2 | - | 0 | 5.90 | 65.59 | 0 |
| No. 3 | - | 0 | 0.30 | 0 | 0 |
| No. 4 | - | 0 | 0 | 0 | 0 |
| No. 5 | - | 0 | 0 | 0 | 0 |
| No. 6 | - | 0 | 0.70 | 0 | 0 |
| No. 7 | - | 0 | 0 | 0 | 0 |
| No. 8 | - | 0 | 2.70 | 82.00 | 38.20 |
| No. 9 | - | 0 | 0.40 | 30.64 | 0 |
| No. 10 | - | 0 | 4.93 | 11.17 | 0 |
| No. 11 | - | 0 | 0 | 0.48 | 0 |
| No. 12 | - | 0 | 1.70 | 0 | 0 |
| No. 13 | - | 0 | 2.89 | 2.61 | 0 |
| No. 14 | 1 | 13 | 9.20 | 65.92 | 0 |
| No. 15 | 1 | 9 | 24.60 | 67.06 | 0 |
| No. 16 | 1 | 8 | 39.80 | 82.80 | 0 |
| No. 17 | 1 | 9 | 37.30 | 74.27 | 0 |
| No. 18 | 1 | 15 | 38.90 | 87.41 | 45.20 |
| No. 19 | 1 | 5 | 74.30 | 83.53 | 81.26 |
| No. 20 | 1 | 5 | 17.20 | 82.40 | 12.81 |
| No. 21 | 1 | 6 | 22.30 | 82.59 | 62.06 |
| No. 22 | 1 | 8 | 13.70 | 0 | 0 |
| No. 23 | 2 | 24 | 54.40 | 72.94 | 0 |
| No. 24 | 2 | 26 | 35.60 | 67.53 | 0 |
| No. 25 | 2 | 18 | 31.10 | 69.12 | 0 |
| No. 26 | 2 | 14 | 42.10 | 38.84 | 0 |
| No. 27 | 2 | 12 | 8.30 | 66.69 | 63.40 |
| No. 28 | 2 | 41 | 24.40 | 93.33 | 69.30 |
| No. 29 | 2 | 11 | 70.50 | 64.11 | 55.30 |
| No. 30 | 2 | 16 | 56.90 | 81.80 | 70.90 |
| No. 31 | 2 | 72 | 27.90 | 56.70 | 52.30 |
| No. 32 | 2 | 22 | 31.90 | 94.25 | 46.70 |
| No. 33 | 2 | 48 | 43.20 | 91.37 | 88.50 |
| No. 34 | 2 | 71 | 37.70 | 86.56 | 66.87 |
| No. 35 | 2 | 6 | 20.00 | 82.07 | 79.08 |
| No. 36 | 2 | 47 | 34.10 | 73.06 | 71.54 |
| No. 37 | 2 | 33 | 38.50 | 78.78 | 66.96 |
| No. 38 | 2 | 13 | 25.10 | 74.94 | 70.06 |
| No. 39 | 2 | 33 | 43.00 | 69.76 | 67.89 |
| No. 40 | 3 | 38 | 28.10 | 0 | 0 |

Cases No. 1-13: hemangiomas; cases No. 14-40: hemangiosarcomas; HPF, high power fields.
