# Supplementary material for: Immunohistochemical detection of p53 and pp53 Ser392 in canine hemangiomas and hemangiosarcomas located in the skin
Source: BMC Vet Res. 2020 Jul 13;16:239. doi: 10.1186/s12917-020-02457-6 (PMC7359283; doi:10.1186/s12917-020-02457-6)
Supplement: Supplementary file 2 — Additional file 2 : Table S2. p53 and pp53 Ser392 indexes in canine visceral hemangiosarcomas. [file 12917_2020_2457_MOESM2_ESM.docx]

**Supplemental Table S2.** p53 and pp53 Ser^392^ indexes in canine visceral hemangiosarcomas

| **Cases** | **p53 index** | **pp53 Ser^392^ index** |
| --- | --- | --- |
|  | **(%)** | **(%)** |
| No. 1 | 21.60 | 0 |
| No. 2 | 32.13 | 0 |
| No. 3 | 31.43 | 0 |
| No. 4 | 28.65 | 12.30 |
| No. 5 | 43.63 | 20.30 |
| No. 6 | 48.77 | 12.90 |
| No. 7 | 43.69 | 0 |
| No. 8 | 0 | 0 |
| No. 9 | 0.01 | 0 |
| No. 10 | 57.30 | 0 |
| No. 11 | 84.64 | 31.50 |
| No. 12 | 87.60 | 41.51 |
| No. 13 | 85.30 | 38.98 |
| No. 14 | 63.01 | 0 |
| No. 15 | 17.71 | 15.20 |
| No. 16 | 29.13 | 0 |
| No. 17 | 70.45 | 0 |
| No. 18 | 39.55 | 29.22 |
